# Mindfulness Meditation Targets Transdiagnostic Symptoms Implicated in Stress-Related Disorders: Understanding Relationships between Changes in Mindfulness, Sleep Quality, and Physical Symptoms

**DOI:** 10.1155/2018/4505191

**Published:** 2018-05-13

**Authors:** Jeffrey M. Greeson, Haley Zarrin, Moria J. Smoski, Jeffrey G. Brantley, Thomas R. Lynch, Daniel M. Webber, Martica H. Hall, Edward C. Suarez, Ruth Q. Wolever

**Affiliations:** ^1^Department of Psychology, College of Science and Mathematics, Rowan University, 201 Mullica Hill Road, Glassboro, NJ 08028, USA; ^2^Thomas Jefferson University, 130 S. 9th Street, Philadelphia, PA 19107, USA; ^3^Department of Psychology and Neurosciences, Duke University, DUMC Box 3026, Durham, NC 27710, USA; ^4^Department of Psychiatry & Behavioral Sciences, Duke University School of Medicine, Box 3022, Durham, NC 27705, USA; ^5^Duke Integrative Medicine, Duke University Medical Center, 3475 Erwin Road, Durham, NC 27705, USA; ^6^School of Psychology, University of Southampton Highfield Campus, Shackleton Building (B44), Southampton SO17 1BJ, UK; ^7^Department of Pathology & Immunology, Washington University School of Medicine, 660 S. Euclid Ave., St. Louis, MO 63110, USA; ^8^Departments of Psychiatry, Psychology, and Clinical and Translational Science, University of Pittsburgh School of Medicine, 3811 O'Hara Street, Room E-1131, Pittsburgh, PA 15213, USA; ^9^Osher Center for Integrative Medicine at Vanderbilt, Department of Physical Medicine and Rehabilitation, Vanderbilt University Medical Center, 3401 West End, Suite 380, Nashville, TN 37203, USA; ^10^Department of Psychiatry & Behavioral Sciences, Vanderbilt University Medical Center, Nashville, TN, USA; ^11^Vanderbilt University School of Nursing, Nashville, TN, USA

## Abstract

Mindfulness-Based Stress Reduction (MBSR) is an 8-week meditation program known to improve anxiety, depression, and psychological well-being. Other health-related effects, such as sleep quality, are less well established, as are the psychological processes associated with therapeutic change. This prospective, observational study (*n* = 213) aimed to determine whether perseverative cognition, indicated by rumination and intrusive thoughts, and emotion regulation, measured by avoidance, thought suppression, emotion suppression, and cognitive reappraisal, partly accounted for the hypothesized relationship between changes in mindfulness and two health-related outcomes: sleep quality and stress-related physical symptoms. As expected, increased mindfulness following the MBSR program was directly correlated with decreased sleep disturbance (*r* = −0.21, *p* = 0.004) and decreased stress-related physical symptoms (*r* = −0.38, *p* < 0.001). Partial correlations revealed that pre-post changes in rumination, unwanted intrusive thoughts, thought suppression, experiential avoidance, emotion suppression, and cognitive reappraisal each uniquely accounted for up to 32% of the correlation between the change in mindfulness and change in sleep disturbance and up to 30% of the correlation between the change in mindfulness and change in stress-related physical symptoms. Results suggest that the stress-reducing effects of MBSR are due, in part, to improvements in perseverative cognition and emotion regulation, two “transdiagnostic” mental processes that cut across stress-related disorders.

## 1. Introduction

Stress-related symptoms and disorders, from anxiety and depression to insomnia and high blood pressure, are highly prevalent and costly to individuals, communities, employers, and the healthcare system [1]. Moreover, stress-related conditions often cluster, resulting in high rates of comorbidity and marked challenges for treatment and prevention [2]. Collectively, stress-related symptoms, including chronic musculoskeletal pain, sleep problems, lethargy and fatigue, depression, anxiety, headache, gastrointestinal complaints, and cardiovascular symptoms, account for the majority of visits to primary care physicians [3]. Thus, unmanaged stress represents a significant burden to individuals and the healthcare system alike. Conventional medical treatments for common stress-related symptoms typically include drugs and brief lifestyle counseling or psychotherapy designed to relieve symptoms [4]. However, pharmacological therapies in particular often fail to address the underlying causes of stress, which are rooted in perception and appraisal [5]. Drawbacks to conventional treatments include financial costs, adverse effects, difficulty with adherence, and limited therapeutic response rates. These practical challenges make stress-related symptoms and comorbid stress disorders difficult to treat efficiently and effectively. Evidence-based complementary and alternative medicine (CAM) options are needed to augment conventional approaches to reducing the burden of stress on health. Mind-body medicine interventions, such as meditation and yoga, represent a participatory medicine approach that integrates behavioral, self-care practices with ongoing conventional care as needed [6].

Mindfulness-Based Stress Reduction (MBSR) is a standardized, 8-week program in mindfulness meditation that was originally designed to help patients manage stress, pain, and illness [7]. Several recent systematic reviews and meta-analyses have documented a consistent effect of MBSR on improving subjective, patient-reported outcomes, including symptoms of stress, anxiety, depression, pain, and quality of life [8–11]. Far less is known, however, about how mindfulness-based interventions (MBIs) like MBSR achieve symptom reduction. The perseverative cognition hypothesis [12] asserts that cognitive perseveration serves to prolong physiological responses to emotional stress. This model articulates a pathway by which chronic cognitive perseveration is linked to ongoing emotional reactions and downstream stress physiology (e.g., cardiovascular, metabolic, neuroendocrine, and immune/inflammatory systems) that ultimately progresses to stress-related chronic disease [13, 14]. Cognitive perseveration can take many forms, including worry, rumination, catastrophizing, or other modes of narrow, self-focused, negative, repetitive thought, whether conscious or unconscious. Emotion regulation strategies are believed to either amplify or buffer this psychophysiological cascade, with important implications for either increasing risk for or developing resilience to stress-related symptoms and disorders. Maladaptive emotion regulation includes some forms of perseverative cognition in which individuals overengage with negative emotions, like ruminating in response to sadness and worrying about unwanted intrusive thoughts, as well as other strategies in which people underengage with unpleasant thoughts, emotions, or physical sensations via experiential avoidance or suppression [15]. These pathological forms of perseverative cognition and maladaptive emotion regulation have been implicated in numerous mental and physical disorders and thus are referred to as “transdiagnostic” processes [16, 17]. Mindful emotion regulation, on the other hand, is characterized by a different way of attending and responding to negative emotions, marked by a sense of focused attention, open awareness, acceptance/nonjudgment, compassion, curiosity, and the ability to consciously act versus automatically react to stress [18, 19]. Given the way in which these core qualities of mindfulness may mitigate and transform maladaptive cognition and emotion regulation processes, mindfulness meditation has been proposed as a “transtherapeutic” approach to transdiagnostic mental processes [20, 21]. Together, the theories of perseverative cognition and mindful emotion regulation provide a conceptual framework for how to understand the clinical outcomes of MBSR, as well as the processes underlying therapeutic change.

**Figure 1:**
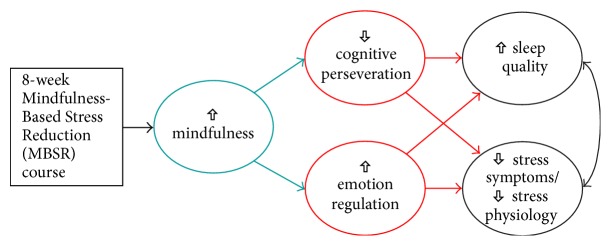
Conceptual model showing hypothesized mechanisms of mindfulness involved in MBSR. Specifically, increased mindfulness is linked to reduced cognitive perseveration (e.g., lower rumination, fewer unwanted intrusive thoughts) and enhanced emotion regulation (e.g., greater cognitive reappraisal, less emotion suppression, less thought suppression, and less avoidance) that, together, may partly explain improved sleep quality and stress-related physical symptoms, both of which are linked to cardiovascular disease (CVD) risk and other chronic health conditions. Adapted from J. M. Greeson (PI) NIH grant application K99AT004945. “Mechanisms of Mindfulness: Effects on Sleep Quality, Stress Physiology & CVD Risk.”

Despite burgeoning evidence for the clinical efficacy of MBSR [8–11], it remains unclear whether changes in mindfulness directly correlate with changes in stress-related symptoms, including physical symptoms of stress, sleep, and transdiagnostic processes. The present study, therefore, aimed to answer three research questions: (1) Is MBSR associated with significant improvements in physical symptoms of stress, sleep quality, and transdiagnostic processes in a community sample of adults? (2) Are such outcomes directly related to increased mindfulness following MBSR? (3) Is the hypothesized link between increased mindfulness and improved symptoms of stress and sleep quality partly explained by changes in transdiagnostic processes that, in theory, are amenable to mindfulness training? Given our review of existing literature, we hypothesized that an 8-week, intensive training program in mindfulness meditation could reduce stress-related symptoms and enhance sleep quality, in part, by targeting two key transdiagnostic processes (perseverative cognition and emotion regulation) that cut across numerous, stress-related mental and physical disorders (see Figure 1). Should evidence support mindfulness training as a “transtherapeutic” approach to reducing transdiagnostic symptoms, then MBSR and other MBIs [22, 23] can be disseminated as a complementary or integrative approach to treating (or preventing) common, chronic, and costly stress-related disorders.

## 2. Materials and Methods

### 2.1. Study Design

The methods of this study have been described in detail elsewhere [24, 25]. Briefly, after providing informed consent, study participants completed standardized self-report questionnaires via the Internet before and after taking an eight-week, self-pay, community MBSR course at a large academic integrative medicine center. The medical center's institutional review board approved the study. MBSR program participants were eligible for the online survey study if they were (1) at least 18 years of age, (2) proficient in English, and (3) able to use a computer with Internet access from home, work, or a public location. To protect against social desirability bias and potential evaluation bias, MBSR course instructors were not directly involved in participant recruitment, consent, or assessment procedures.

### 2.2. Participants

Participants included 322 individuals enrolled in a community MBSR program who completed a pre-MBSR survey. Of those who enrolled, 213 provided data on a post-MBSR survey, a response rate of 66%. As reported in a previous analysis of depressive symptom outcomes from this same study [25], participants were primarily well-educated, white women with a mean (SD) age of 45 (12) who were married and working full-time. Nearly two-thirds of the sample reported affiliation with a religion, primarily Christianity. Over half of the current study participants (55%) were at risk for depression based on high rumination scores (Ruminative Responses Scale [RRS] > 45) [26]. Approximately half of the study sample met cutoff criteria for a “likely case” of clinical depression or anxiety based on a Hospital Anxiety and Depression Scale (HADS) subscale score > 8 [27]. In addition, over half (57%) of study participants reported poor sleep based on a Pittsburgh Sleep Quality Index (PSQI) global score > 5 [28]. Consistent with these self-reported psychological symptoms, the most common motivations for taking the MBSR program were to improve mental health (90%), to help manage stress (89%), for personal growth and self-discovery (81%), and to improve physical health (61%). Over half the sample (58%) also reported prior experience with contemplative practice (i.e., meditation, mindfulness, or contemplative prayer), with a median less than 1 year of experience. Taken together, the study sample reported experiencing clinically relevant symptoms of anxiety, depression, rumination, and poor sleep quality prior to undertaking the MBSR course.

### 2.3. Procedure

Study participants were surveyed within 1 week before the first MBSR class session and again within 1 week after the last MBSR class. The secure, online survey was administered by using ViewsFlash software (Cogix, Monterey, CA) and included basic demographic information and a battery of standardized self-report questionnaires. Among other psychosocial variables, the questionnaires assessed dispositional (trait) mindfulness, along with symptoms of stress, cognitive perseveration, emotion regulation, and sleep quality [24, 25]. Participants were offered compensation ($10) for completing the surveys after grant funding was obtained.

### 2.4. Intervention

The intervention followed a standard 8-week MBSR course based on the work of Kabat-Zinn [7]. Courses were taught by highly experienced instructors with an average of 13 years (range, 10–20 years) teaching MBSR and a minimum of 7 days of professional education and training coordinated by the Center for Mindfulness in Medicine, Health Care, and Society (Worcester, MA). In addition, when hired, MBSR instructors had a minimum of 3 years of personal experience with mindfulness meditation, including at least two extended teacher-led retreats in mindfulness (Vipassana) meditation. As part of the MBSR program, participants were instructed to practice 20–45 minutes of formal meditation daily, 6 days per week, in addition to the informal practice of being mindful during everyday activities. Weekly class time lasted 2.5 hours. Additionally, the course included 1 full-day (7-hour) meditation retreat on the weekend of the sixth week. Written materials and audio CDs of guided mediations and yoga were provided to support home practice.

### 2.5. Measures

*Cognitive and Affective Mindfulness Scale-Revised (CAMS-R)*. The 12-item CAMS-R [19] was used to measure four core qualities of mindfulness: stable attention, awareness of thoughts and feelings, acceptance/nonjudgment, and present-moment focus. Items are rated on a 4-point scale from “rarely/not at all” to “almost always.” Scores range from 12 to 48. Previous psychometric evaluation of the CAMS-R found the instrument to be reliable and valid [19]. Further, prior analyses from the present study found that the CAMS-R was sensitive to change following 8 weeks of mindfulness meditation training [24, 25].

*Cohen-Hoberman Inventory of Physical Symptoms (CHIPS)*. The CHIPS [29] is a list of 33 common physical symptoms. Items were carefully selected so as to exclude symptoms of an obviously psychological nature (e.g., felt nervous or depressed). The scale does, however, include many physical symptoms that have been traditionally viewed as psychosomatic (e.g., headache, constant fatigue, muscle tension, indigestion, and weight loss). Each item is rated for how much that problem bothered or distressed the individual during the past two weeks. Items are rated on a 5-point scale from “not at all” to “extremely.” Scores range from 0 to 123. Higher scores reflect greater perceived burden due to stress-related physical symptoms and positively correlate with healthcare utilization [29]. 

*Pittsburgh Sleep Quality Index (PSQI)*. The PSQI is a 19-item self-report scale that measures sleep quality across seven different domains (subjective sleep quality, sleep latency, sleep duration, sleep efficiency, sleep disturbance, use of sleep medication, and daytime dysfunction). A global score is calculated, with higher scores indicating greater sleep disturbance and lower scores better sleep quality. A total score above 5 has been shown to discriminate poor sleepers (diagnosed with insomnia) from good (normal) sleepers [28]. Sleep quality, along with the remaining measures listed below, has been identified as a transdiagnostic symptom, associated with increased risk of numerous mental and physical health disorders [30, 31]. 

*Ruminative Responses Scale (RRS)*. The RRS is a 22-item scale from the Response Styles Questionnaire that uses a 4-point Likert type scale to assess ruminative coping responses to depressed mood (e.g., “I think about why cannot I handle things better” and “I think about how sad I feel”). Scores range from 22 to 88. Higher RRS scores indicate greater levels of self-focused perseverative cognition and have been found to predict worse depression symptom severity. A score over 45 has been associated with increased risk of clinical depression [28]. 

*White Bear Suppression Inventory (WBSI)*. The WBSI [32] is a 15-item questionnaire designed to measure thought suppression. Items are rated on a 5-point Likert scale from “strongly disagree” (1) to “strongly agree” (5). The total score ranges from 15 to 75. Higher scores reflect a greater tendency to suppress thoughts and are associated with greater severity of anxiety and depressive symptoms and with higher autonomic arousal. Two subscales are generated: unwanted intrusive thoughts (UIT), an indicator of perseverative cognition, and thought suppression (TS), a compensatory cognitive emotion regulation strategy. 

*Acceptance and Action Questionnaire (AAQ-9)*. The 9-item version of the AAQ [33] measures “experiential avoidance.” Avoidance in this case indicates the tendency to avoid or alter negative thoughts, feelings, and physiological sensations, even when doing so interferes with taking effective action like pursuing valued goals. Prior studies have found that higher AAQ-9 scores correlate with higher stress-related psychological and physical symptoms [33]. The AAQ-9 was included in the present study, as mindfulness meditation practice intentionally cultivates acceptance, rather than avoidance, of negative thoughts, feelings, and physical sensations, which would be reflected in lower AAQ scores. Because the survey battery was trimmed during the first year of the study to reduce participant burden, the AAQ was only available for 71 MBSR completers. 

*Emotion Regulation Questionnaire (ERQ)*. The ERQ [34] is a 10-item questionnaire that is designed to assess individual differences in the habitual use of two emotion regulation strategies: cognitive reappraisal of stress and negative emotions (6 items; e.g., “when I'm faced with a stressful situation, I make myself think about it in a way that helps me stay calm”) and expressive suppression (4 items; e.g., “I keep my emotions to myself”). Items are scored using a 7-point Likert scale (strongly disagree = 1 and strongly agree = 7). Higher cognitive reappraisal scores and lower expressive suppression scores have been associated with better psychological well-being [34].

All measures of perseverative cognition (RRS, WBSI-UIT) and emotion regulation (WBSI-TS, AAQ-9, ERS-suppression, and ERQ-reappraisal) have been identified as “transdiagnostic” processes that cut across numerous stress-related psychological and medical disorders; as such, these variables represent an important intervention target and a viable mechanism of therapeutic change for mindfulness-based interventions [20].

### 2.6. Data Analysis

Descriptive statistics were performed using SPSS software, version 24 (IBM, Armonk, NY). Variables were screened for distributional assumptions before analysis. All continuous variables approximated a normal distribution with skewness and kurtosis values less than 3.0. Preliminary analyses (independent samples *t*-tests) were used to test for baseline differences between post-MBSR survey completers and noncompleters.

Paired *t*-tests were performed to assess the statistical significance of pre-post MBSR changes in mindfulness, stress symptoms, sleep quality, and transdiagnostic measures of cognitive perseveration and emotion regulation. Effect sizes for pre-post changes were calculated using Cohen's *d* for paired samples; specifically, the pre-post difference score was divided by the SD of the pre-post difference scores [35]. Cohen's *d* values can be interpreted as *d* = .20 is a small effect, *d* = .50 is a medium sized effect, and *d* = .80 or higher is a large effect. Small magnitude effects are meaningful in behavioral sciences, whereas effect sizes below *d* = .20 are considered negligible [35].

Bivariate correlations were used to test for direct associations between MBSR-related changes in mindfulness, stress symptoms, sleep quality, and transdiagnostic variables. Partial correlations were then run to test whether hypothesized associations between changes in mindfulness and changes in stress symptoms and sleep quality, respectively, remained significant when controlling for changes in each transdiagnostic outcome measure. Diminished correlation coefficients and *p* values in partial correlation analyses were interpreted as support for the overarching hypothesis that improvement in transdiagnostic measures of perseverative cognition and emotion regulation partly accounted for the relationship between increased mindfulness and decreased stress-related symptoms and sleep disturbance following MBSR training.

Lastly, seven covariates were added to the partial correlation analyses to test whether observed associations were independent of demographic characteristics (age, gender, education, household income, and employment status), as well as prior meditation experience (yes/no) and expectation to benefit from the MBSR course (none, a little bit, somewhat, quite a bit, and a great deal), all of which could potentially confound individual differences in psychological symptoms and responsiveness to the MBSR program.

Alpha was set at *p* = 0.05 (2-tailed) for all statistical tests performed.

## 3. Results and Discussion

### 3.1. Preliminary Analyses

As previously reported on this sample [25], participants who completed the post-MBSR survey had lower depressive symptom severity at baseline. In addition, for the present analyses, we found that participants who did not complete the post-MBSR survey had slightly lower trait mindfulness scores at baseline (completers = 28.50, noncompleters = 29.86, *p* = 0.041), higher scores on unwanted intrusive thoughts (completers = 28.68, noncompleters = 31.08, *p* = 0.013), and higher scores on emotion suppression (completers = 3.14, noncompleters = 3.70, *p* < 0.001). Post-MBSR survey completers did not differ at baseline on other measures, including anxiety, rumination, sleep quality, physical symptoms of stress, thought suppression, avoidance, or cognitive reappraisal.

### 3.2. Main Results

As shown in Table 1, *t*-tests revealed statistically significant improvements for all outcome measures. Effect sizes were large for the change in mindfulness, medium for changes in sleep quality, stress symptoms, rumination, unwanted intrusive thoughts, thought suppression, avoidance, and cognitive reappraisal and small for change in expressive suppression.

As expected, bivariate correlations showed a significant, direct association between increased mindfulness and decreased stress-related physical symptoms (*r* = −0.384, *p* < 0.001) and increased sleep quality (*r* = −0.211, *p* = 0.004), respectively (Table 2). In addition, increased mindfulness was directly correlated with improvement in perseverative cognition and emotion regulation, including decreased rumination, unwanted intrusive thoughts, thought suppression, experiential avoidance, and expressive suppression, as well as increased cognitive reappraisal (see Table 2). Decreased stress-related physical symptoms were directly correlated with increased sleep quality, and both of these main outcomes correlated significantly with changes in each of the transdiagnostic processes in the expected direction (see Table 2).

When controlling for changes in transdiagnostic processes, partial correlation analyses revealed somewhat lower correlation coefficients between changes in mindfulness and changes in stress-related physical symptoms (Table 3). For example, whereas the bivariate correlation between change in mindfulness and change in physical stress symptoms was −0.384  (*p* < 0.001), controlling for change in rumination lowered the partial correlation to *r* = −0.292, a reduction of 0.092 points. Thus, controlling for change in rumination accounted for 24% (0.092 points) of the bivariate association between change in mindfulness and change in physical symptoms of stress. Even after controlling for rumination, the *p* value remained unchanged (*p* < 0.001), demonstrating that the original association remained significant. This same pattern held true for intrusive thoughts and thought suppression, while emotion suppression and cognitive reappraisal showed somewhat weaker effects, and avoidance did not appear to change the correlation coefficient at all (see Table 3). Specifically, the original bivariate correlation between change in mindfulness and change in physical symptoms of stress dropped from *r* = −0.384 to between −0.267 (when controlling for intrusive thoughts) and −0.371 (when controlling for avoidance). Each transdiagnostic process accounted for between 3% and 30% of the original bivariate correlation, and all *p* values for partial correlations involving stress-related physical symptoms remained highly statistically significant (*p* < 0.001). Further controlling for demographic covariates did not substantively alter the partial correlation coefficients, nor the *p* values.

A similar pattern was observed for partial correlation analyses involving changes in mindfulness and sleep quality (Table 4). Overall, changes in transdiagnostic processes accounted for a relatively larger proportion of the original bivariate correlation, between 16% and 32%. For example, when controlling for reappraisal, the original bivariate correlation and corresponding *p* value dropped from *r* = −0.211  (*p* = 0.004) to −0.178  (*p* = 0.015). Similar effects were observed for rumination, intrusive thoughts, and thought suppression. When controlling for change in avoidance, the original bivariate correlation fell even more, to −0.144  (*p* = 0.28), and became nonsignificant. Further controlling for the 7 demographic covariates pushed four partial correlations (involving rumination, intrusive thoughts, thought suppression, and emotion suppression, resp.) slightly over the *p* = 0.05 threshold. In contrast, adding covariates to the partial correlation that controlled for avoidance doubled the correlation coefficient from *r* = −0.144 to −0.286. This indicated that the association between increased mindfulness and increased sleep quality following MBSR actually strengthened and again became statistically significant when covariates were added to the partial correlation model for avoidance.

### 3.3. Discussion

In summary, this study examined the degree to which changes in perseverative cognition and emotion regulation partly explain the link between increased mindfulness and improved stress symptoms and sleep quality following an 8-week course in mindfulness meditation. Results replicated pre-post changes in mindfulness and other self-reported psychosocial outcomes documented in recent systematic reviews and meta-analyses [8–11, 36, 37]. Our findings extend prior work by documenting direct correlations between changes in mindfulness and improvements in physical symptoms of stress and sleep quality. Furthermore, these findings begin to elucidate the contribution of changes in transdiagnostic processes as candidate mediators underlying therapeutic change. These findings suggest that mindfulness training can effectively target transdiagnostic symptoms that increase the risk of numerous stress-related disorders, making mindfulness-based interventions, like MBSR, a promising approach to myriad stress-related disorders.

The magnitude of associations linking increased mindfulness and improved stress symptoms and sleep quality remained significant after controlling for the transdiagnostic processes. Hence, there are clearly additional mechanisms that explain how increased mindfulness contributes to improved stress-related mental and physical symptoms. Future studies are needed to investigate other potential psychological, biological, behavioral, and even social variables that might contribute to this explanation. For instance, in the context of depression, changes in self-compassion appear to mediate therapeutic change [38], whereas, in addiction, therapeutic change appears to be partly accounted for by changes in craving, attentional bias, and autonomic regulation measured by heart-rate variability [39, 40]. Recent work also suggests that it would be useful to consider the contribution of personality differences. For example, the positive effects of mindfulness practice may vary depending on whether a person has a primarily undercontrolled (dramatic, erratic, and impulsive) style versus an overcontrolled personality style (constrained emotional expressions, hyper-detail-focused, and overly planful) [41]. Finally, from a transdiagnostic perspective, and as suggested by multiple theories spanning perseverative cognition, emotion regulation, and the reactivity hypothesis, changes in objective behavioral or biological markers of stress and self-regulation represent another important direction to pursue.

The current study had several limitations that merit discussion. First, we included a self-selected community sample of adults that had already registered for a self-pay MBSR course at a large academic medical center and who volunteered to take part in research for either no compensation or very modest financial compensation. Although the demographic profile of this cohort (i.e., primarily well-educated, white women who were working full-time) was similar to other published studies of well-regarded self-pay MBSR programs at comparable institutions [42, 43], it is possible that there could be a selection bias, such that positive expectations may have conflated outcomes, and/or participants' reported outcomes may not generalize to a more diverse demographic constituency. We tried to mitigate this by controlling for expectations along with demographics. Second, there is some risk of attrition bias, as 1/3 of those enrolled in the study did not complete the post-MBSR survey. The 2/3 response rate, however, is comparable to open trials in similar academic medical settings [43–45]. In addition, our findings on select baseline differences parallel those from at least one other program [43] in regard to attrition. Whereas Gawrysiak and colleagues found that higher baseline stress levels and lower positive effect were significantly associated with attrition, we found that individuals who were missing postintervention data had higher scores for depression symptoms, intrusive thoughts, and emotion suppression and lower scores for mindfulness at baseline. Fortunately, our large sample size afforded adequate statistical power, and the demographic profile of this sample reflected the demographics reported at other large community MBSR programs at peer academic medical centers, lending confidence to the findings. We also note that comparing mean baseline scores, particularly with relatively large sample sizes, can produce statistically significant differences that are unlikely to be clinically meaningful. For example, the mean baseline CAMS-R score only differed by about one point between completers and noncompleters, and completers did not differ on baseline measures of anxiety, sleep quality, physical symptoms of stress, avoidance, thought suppression, cognitive reappraisal, or rumination (a risk factor for future depression). Beyond considering statistical versus clinical significance in large samples, we also offer a practical suggestion for future studies. MBSR instructors and program evaluators should consider using weekly meditation diaries to assess for possible struggles with mindfulness, stress levels, depressive symptoms, unwanted intrusive thoughts, and the tendency to suppress emotional expression. Doing so may afford instructors an opportunity to respond to participants by providing personalized support and practice suggestions, which, in turn, may help mitigate attrition and maximize data collection, while potentially optimizing clinical outcomes. Finally, it is possible that observed effects may or may not be robust across demographic subgroups or different levels of prior experience with meditation. As with positive expectancy, we tried to adjust for these potential concerns by including demographic characteristics and prior meditation experience as covariates. Results for stress-related physical symptoms remained unchanged after adding covariates, and though some *p* values decreased in partial correlations for sleep quality, the absolute values of correlation coefficients and *p* values did not change markedly, with the one exception of change in avoidance. Considering the balance of strengths and limitations together, potential threats to validity were identified and addressed, and results are applicable to a major demographic known to pursue MBSR. Additional studies are needed to replicate these findings in other MBSR programs that serve economically, racially, and culturally diverse patients and communities.

## 4. Conclusions

Results suggest that the stress-reducing effects of MBSR may be due, in part, to improved perseverative cognition and emotion regulation, two “transdiagnostic” mental processes that cut across stress-related disorders. These findings are important, as they offer empirical evidence to support the overarching hypothesis that mindful emotion regulation may be a key psychological process that contributes to physical and mental health outcomes associated with mindfulness meditation. Such evidence is relevant to better understanding how mindfulness meditation works to target stress-related symptoms, including sleep quality. In addition, these findings implicate a number of cognitive and emotional processes (i.e., rumination, intrusive thoughts, thought suppression, avoidance, emotion suppression, and cognitive reappraisal) as unique contributors to therapeutic change in MBSR programs. Because this pre-post observational study cannot establish causation, experimental studies are needed to determine (a) whether changes in transdiagnostic processes formally mediate stress-related health outcomes in MBSR, (b) what the relative importance is for different transdiagnostic processes in predicting a given outcome, such as depression, anxiety, or high blood pressure, and (c) if changes in transdiagnostic processes operate similarly or differently as a function of either clinical diagnosis or demographic subgroup (e.g., age, gender, race, socioeconomic status, cultural background, and religious affiliation). Answers to these questions will not only help advance our mechanistic understanding of MBSR as a complementary therapy, but will also elucidate who is most likely to benefit from mindfulness training and why.

## Figures and Tables

**Table 1:** Pre-post changes and effect sizes for MBSR outcome measures.

Outcome measure	Pre mean (SD)	Post mean (SD)	Change mean (SD)	df	*t*	*p* value	*d*
Mindfulness (CAMS-R)	29.86 (5.71)	35.22 (5.18)	5.36 (5.14)	212	15.21	<0.001	1.04
Stress symptoms (CHIPS)	20.59 (14.97)	14.13 (11.21)	−6.46 (11.32)	198	8.06	<0.001	0.57
Sleep quality (PSQI)	6.45 (3.77)	5.28 (3.28)	−1.17 (2.80)	186	5.67	<0.001	0.42
Rumination (RRS)	46.91 (10.80)	40.71 (8.54)	−6.20 (9.15)	200	9.61	<0.001	0.68
Unwanted intrusive thoughts (WBSI)	28.74 (8.02)	24.72 (7.04)	−4.02 (6.17)	203	9.31	<0.001	0.65
Thought suppression (WBSI)	18.53 (5.24)	16.54 (4.34)	−2.00 (4.06)	203	7.02	<0.001	0.49
Experiential avoidance (AAQ-9)	32.90 (8.34)	28.75 (7.58)	−4.15 (6.54)	70	5.35	<0.001	0.63
Expressive suppression (ERQ)	3.12 (1.26)	2.83 (1.03)	−0.29 (1.04)	202	4.02	<0.001	0.28
Cognitive reappraisal (ERQ)	4.48 (1.14)	5.16 (0.84)	0.68 (1.11)	202	8.78	<0.001	0.62

**Table 2:** Bivariate correlations for MBSR-related pre-post change scores.

Measures	(1) CAMS-R	(2) CHIPS	(3) PSQI	(4) RRS	(5) WBSI-UIT	(6) WBSI-TS	(7) AAQ	(8) ERQ-SUPP	(9) ERQ-REAP
(1) Mindfulness (CAMS-R)	1								
(2) Physical symptoms of stress (CHIPS)	−0.384^*∗∗∗*^	1							
(3) Sleep quality (PSQI)	−0.211^*∗∗*^	0.362^*∗∗∗*^	1						
(4) Rumination (RRS)	−0.320^*∗∗∗*^	0.400^*∗∗∗*^	0.200^*∗∗*^	1					
(5) Intrusive thoughts (WBSI-UIT)	−0.482^*∗∗∗*^	0.338^*∗∗∗*^	0.171^*∗*^	0.510^*∗∗∗*^	1				
(6) Thought suppression (WBSI-TS)	−0.449^*∗∗∗*^	0.264^*∗∗∗*^	0.163^*∗*^	0.426^*∗∗∗*^	0.635^*∗∗∗*^	1			
(7) Experiential avoidance (AAQ-9)	−0.366^*∗∗*^	0.338^*∗∗*^	0.379^*∗∗*^	0.569^*∗∗∗*^	0.496^*∗∗∗*^	0.568^*∗∗∗*^	1		
(8) Expressive suppression (ERQ)	−0.282^*∗∗∗*^	0.205^*∗∗*^	0.240^*∗∗*^	0.174^*∗*^	0.193^*∗∗*^	0.205^*∗∗*^	0.474^*∗∗∗*^	1	
(9) Cognitive reappraisal (ERQ)	0.235^*∗∗*^	−0.248^*∗∗∗*^	−0.167^*∗*^	−0.197^*∗∗*^	−0.203^*∗∗*^	−0.108	−0.434^*∗∗∗*^	−0.162^*∗*^	1

*Note.* CAMS-R: Cognitive and Affective Mindfulness Scale-Revised. CHIPS: Cohen-Hoberman Inventory of Physical Symptoms. PSQI: Pittsburgh Sleep Quality Index. RRS: Ruminative Responses Scale. WBSI: White Bear Suppression Inventory. UIT: unwanted intrusive thoughts. TS: thought suppression. AAQ-9: Acceptance and Action Questionnaire. ERQ: Emotion Regulation Questionnaire; ^*∗*^*p* < 0.05, ^*∗∗*^*p* < 0.01, and ^*∗∗∗*^*p* < 0.001.

**Table 3:** Partial correlations between change in mindfulness (CAMS-R) and change in physical symptoms of stress (CHIPS).

Controlled transdiagnostic variable	Partial correlation	Partial correlation with covariates
Change in rumination		
*r*	−0.292	−0.296
*p* value	<0.001	<0.001
df	195	188
Change in intrusive thoughts		
*r*	−0.267	−0.272
*p* value	<0.001	<0.001
df	195	188
Change in thought suppression		
*r*	−0.307	−0.303
*p* value	<0.001	<0.001
df	195	188
Change in avoidance		
*r*	−0.371	−0.361
*p* value	0.002	0.004
df	68	61
Change in cognitive reappraisal		
*r*	−0.346	−0.343
*p* value	<0.001	<0.001
df	196	188
Change in expressive suppression		
*r*	−0.348	−0.343
*p* value	<0.001	<0.001
df	196	188

*Note*. Bivariate correlation between change in mindfulness and change in stress symptoms was *r* = −0.384, df = 198, *p* < 0.001. All partial correlations were lower, indicating that each transdiagnostic variable uniquely accounted for part of the original association between increased mindfulness and decreased stress-related physical symptoms. After accounting for changes in transdiagnostic variables and covariates, the correlation between change in mindfulness and change in stress-related symptoms remained statistically significant.

**Table 4:** Partial correlations between change in mindfulness (CAMS-R) and change in sleep quality (PSQI).

Controlled transdiagnostic variable	Partial correlation	Partial correlation with covariates
Change in rumination		
*r*	−0.158	−0.144
*p* value	0.032	0.055
df	183	176
Change in intrusive thoughts		
*r*	−0.149	−0.142
*p* value	0.043	0.059
df	183	176
Change in thought suppression		
*r*	−0.155	−0.140
*p* value	0.035	0.063
df	183	176
Change in avoidance		
*r*	−0.144	−0.286
*p* value	0.282	0.042
df	56	49
Change in cognitive reappraisal		
*r*	−0.178	−0.161
*p* value	0.015	0.032
df	184	176
Change in expressive suppression		
*r*	−0.151	−0.129
*p* value	0.039	0.085
df	184	176

*Note*. Bivariate correlation between change in mindfulness and change in sleep quality was *r* = −0.211, df = 186, *p* = 0.004. All partial correlations were lower, indicating that each transdiagnostic variable uniquely accounted for part of the original association between increased mindfulness and increased sleep quality. After accounting for changes in transdiagnostic variables, the correlation between change in mindfulness and change in stress-related symptoms remained statistically significant, except for change in avoidance, which was measured in fewer cases. Further controlling for covariates did not substantively change the magnitude of partial correlations nor the p values; however, four *p* values become marginally significant, and one (for change in avoidance) dropped markedly.
